# How I do it: simple ad-hoc pulsatile pump model for realistic microsurgical training under pulsatile flow

**DOI:** 10.1007/s00701-025-06608-2

**Published:** 2025-08-13

**Authors:** Richard Parvin, Victor Gabriel El-Hajj, Victor E. Staartjes, Luca Ricciardi, Marisa Gandia-Gonzalez, Pablo Garcia Feijoo, Mateo Tomas Farina Núñez

**Affiliations:** 1 Neuro- and Spine Center, Hirslanden Klinik St. Anna, St. Annastrasse 32, Lützelmatt 3, 6006 Lucerne, Switzerland; 2 Department of Clinical Neuroscience, Karolinska Institutet, Stockholm, Sweden; 3 Machine Intelligence in Clinical Neuroscience & Microsurgical Neuroanatomy (MICN) Laboratory, Department of Neurosurgery, Clinical Neuroscience Center, University Hospital Zurich, University of Zurich, Zurich, Switzerland; 4 Department of NESMOS, Azienda Ospedaliera Universitaria Sant’Andrea, Sapienza University, Rome, Italy; 5 Department of Neurosurgery, University Hospital La Paz, Madrid, Spain; 6 Department of Spine Surgery, Schulthess Klinik, Zurich, Switzerland

**Keywords:** Realistic, Pulsatile pump, Microsurgical training, Microsurgery, Education

## Abstract

**Background:**

This simple pulsatile pump model (PPM) provides a realistic and low-cost model for microsurgical training. In this manner, the use of live animals for realistic microsurgical training is reduced, as it is possible to combine our model with several artificial microsurgical training models while retaining pulsatile flow.

**Method:**

We detail steps for construction of the PPM for realistic microsurgical training under pulsatile flow – as well as microsurgical training examples – in a tried-and-true, cost-effective fashion, from readily available monitoring and infusion materials. The PPM is prepared by assembling a closed-loop system with two pressure applicators (infusion pumps/infusomats), a monitoring set connected to a transducer and display, and various infusion components (two intravenous drip/intrafix sets, saline, three-way stopcocks/discofix C, Heidelberger extension line, and pressure bags), allowing for adjustable pulsation frequency, flow and pressure as well as continuous pressure monitoring. The PPM can be used for a variety of microsurgical tasks, including microvascular repair and anastomosis and aneurysm clipping, providing a realistic and controlled animal-free training environment.

**Conclusion:**

This model enables near-realistic microsurgical training under adjustable pulsatile flow based on readily available materials.

**Supplementary information:**

The online version contains supplementary material available at https://doi.org/10.1007/s00701-025-06608-2.

## Key Points

1. When preparing the pulsatile pump model (PPM), there is no specific caution necessary unless animal vessels or placenta models are used as recipients, in which case this requires appropriate hygiene measures. Materials needed: two adjustable infusion pumps (for example Infusomat Space, B. Braun, Melsungen, Germany), one transducer for continuous pressure monitoring (usually for an arterial line or central venous line, for example Combitrans, B. Braun, Melsungen, Germany), one monitor for displaying the pressure and pulsatile rate, one monitoring set, three bags of saline or Ringer’s solution (500 mL or 1000 mL size), two intravenous infusion/drip sets (for example Intrafix set, B. Braun, Melsungen, Germany), one additional three-way stopcock (for example Discofix C, B. Braun, Melsungen, Germany), a syringe of 18G size, a Heidelberger extension line, and two pressure cuffs (Fig. 1).

**Fig. 1:**
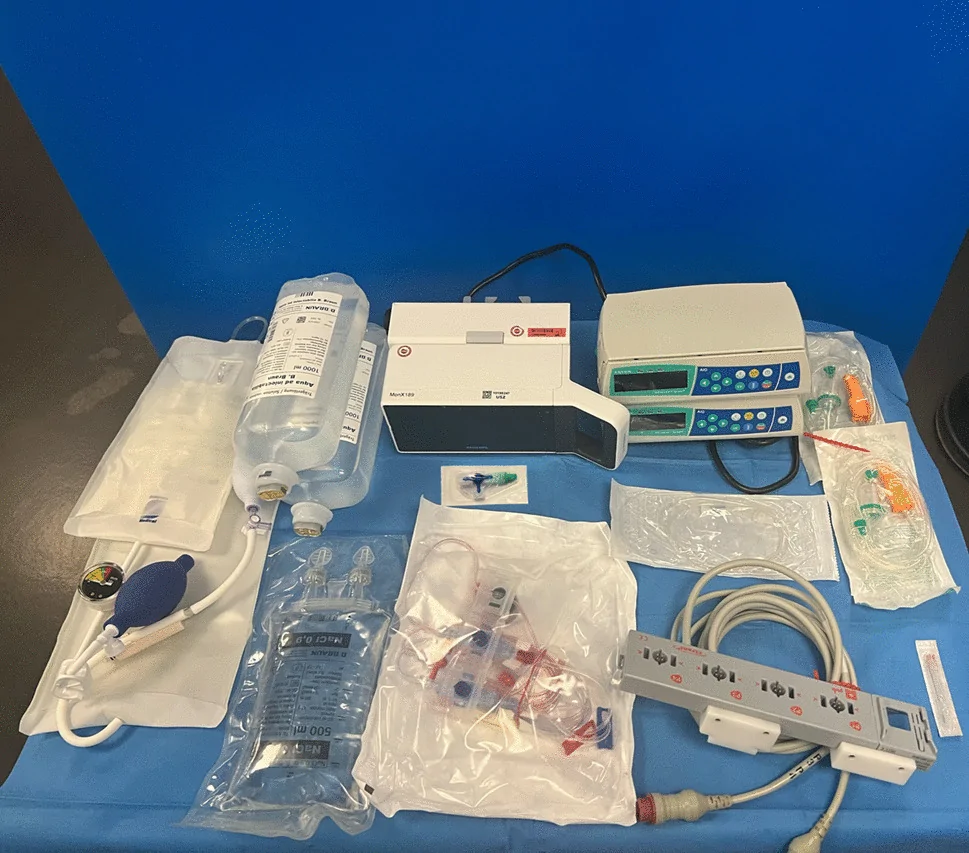
Overview of necessary materials for the pulsatile pump model
2. We prepare the monitoring set in regular fashion by flushing it, as it is performed also for blood pressure monitoring using arterial or central venous catheters (in the video we used 1000 ml aqua ad inject, of course any fluid can be used) and one pressure cuff which is inflated – this being *Bag B* (Fig. 2)

**Fig. 2:**
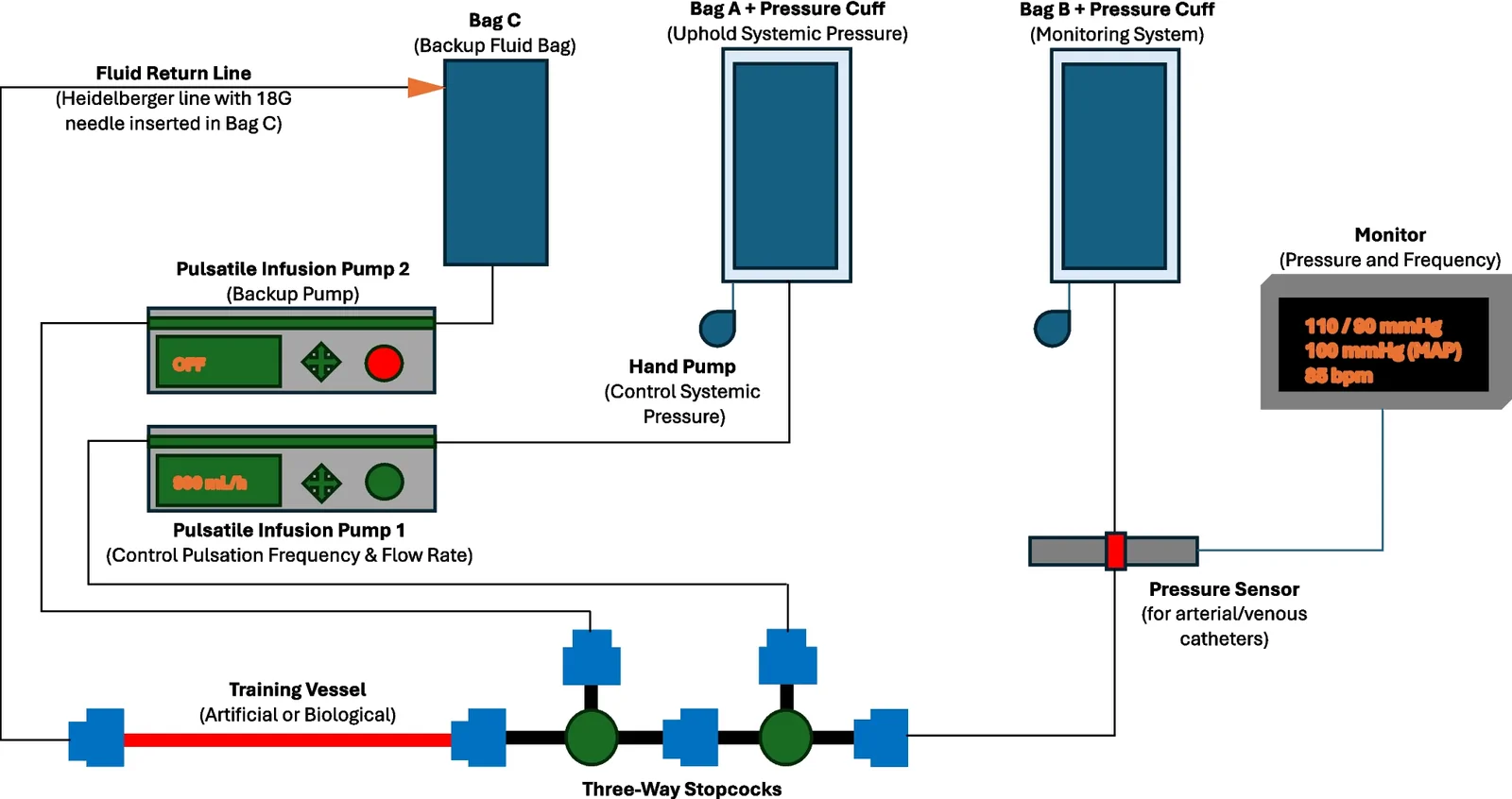
Schematic overview of the pulsatile pump model setup
3. We connect the monitoring set to the proximal part of the three-way stopcock of the monitoring set itself, and to the monitor. The distal end of the stopcock setup is connected to the training vessel, which can either be a synthetic or a biological (e.g. placenta, coronary, or other vessel) vessel via a connector and ligated. Alternatively, the distal end of the stopcock setup can also be inserted into the training vessel using a small-bore Foley catheter – the inflated balloon of the catheter securing it in place.
4. Now, we prepare the second fluid bag (*Bag A*), with its primary purpose being to uphold systemic pressure. Again, we use e.g. 500 ml or 1000 mL 0.9% saline or Ringer’s solution bag put into a pressure cuff. The bag is then connected to an intravenous infusion set and then connected directly to the adjustable *infusion pump 1* (**as demonstrated again in **Fig. 2). When turned on, this infusion pump adds pulsatility and controlled flow to the system. The end of this line is then connected to the three-way stopcock.
5. A third fluid bag (*Bag C*) connected to an intravenous infusion set and then connected via a second adjustable infusion pump (*pulsatile infusion pump 2*) to the stopcocks. This second infusion pump remains turned off for now. From the distal end of the training vessel, a Heidelberger extension line (this being our fluid return line) is connected to a 18G needle which is inserted into *bag C*. In this way, during microsurgical training under continuous pulsatile flow, *bag C* (being connected to infusion pump 2) is filled up.
6. After all is now set up, the system pressure can be set using the hand pump on *bag A.* Pressures from around 100 mmHg to 200 mmHg can be easily set. In our experiments, pressures set using the hand pump correlated well with the mean arterial pressure (MAP) measured through the monitoring system.
7. Control over pulsation frequency and flow rate is achieved by turning on pump 1. A setting of flow rate of 600 mL/h equals approximately a frequency of 60 bpm, 900 mL/h approximately 90 bpm, and 1200 mL/h approximately 120 bpm using our setup (Fig. 3). The pulse width is around ± 10 mmHg around the MAP value.

**Fig. 3:**
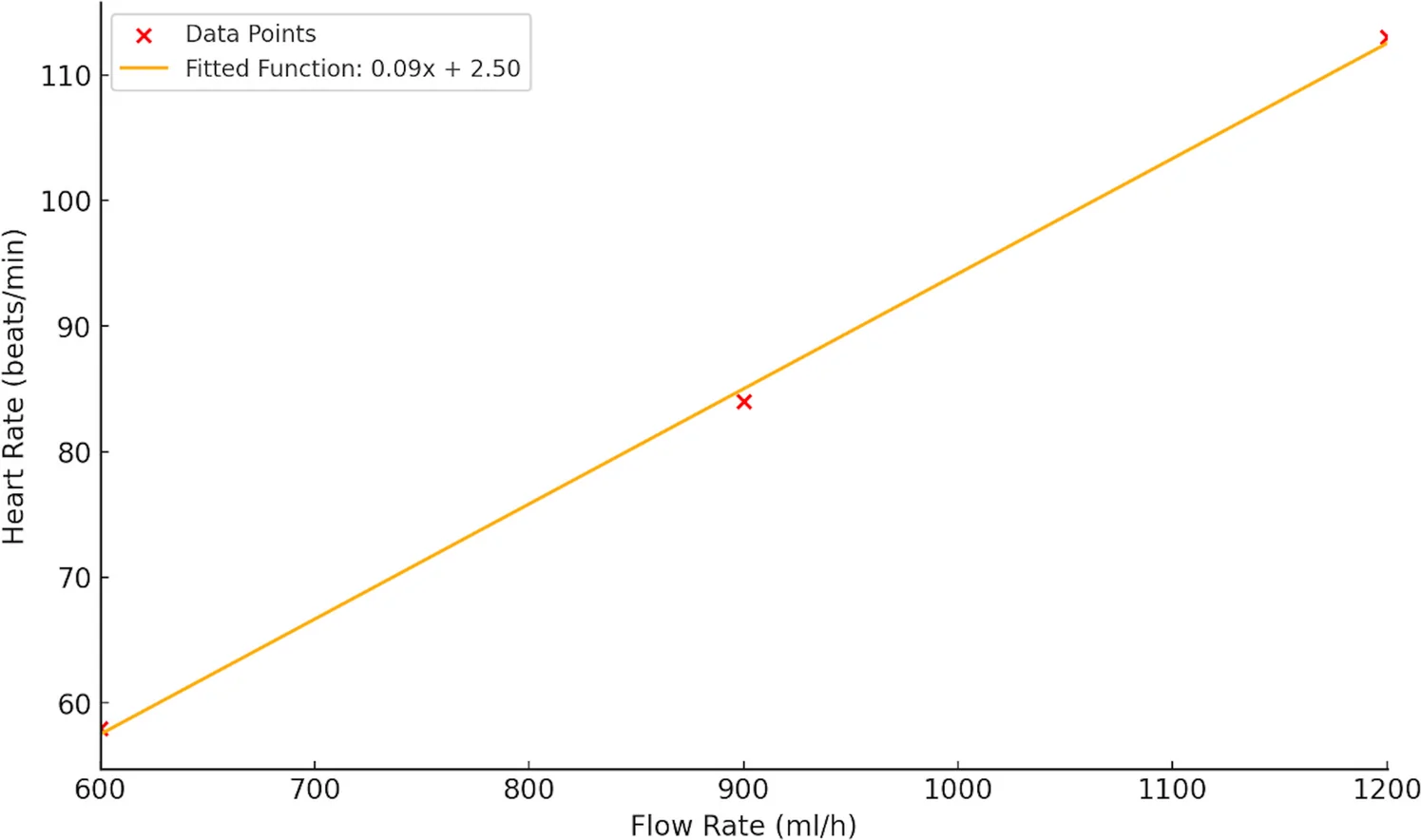
Fitted linear function demonstrating the relationship of pulsation frequency (heart rate) that is achieved in our setup depending on which flow rate is chosen for the infusion pump (Infusomat Space in our case)
8. Once *bag A* depletes, the setup can then simply be alternated between the two *bags A and C* by exchanging the pressure cuff onto *bag C*, and by using the other infusion pump (*Nr. 2*) and connecting the fluid return line to *bag A*. This switching manoeuvre can be performed within about a minute. Creating a closed circle is important for using the same solutions repeatedly.
9. This system can be used for a) microvascular repair, b) end-to-end anastomosis, c) end-to-side anastomosis, d) microsurgical clipping, etc., and provides a pulsatile realistic simulation of blood flow dynamics, enabling the assessment of surgical techniques, the evaluation of suture patency, and the training of surgeons under conditions mimicking physiological blood pressure and continuous flow (Fig. 4).

**Fig. 4:**
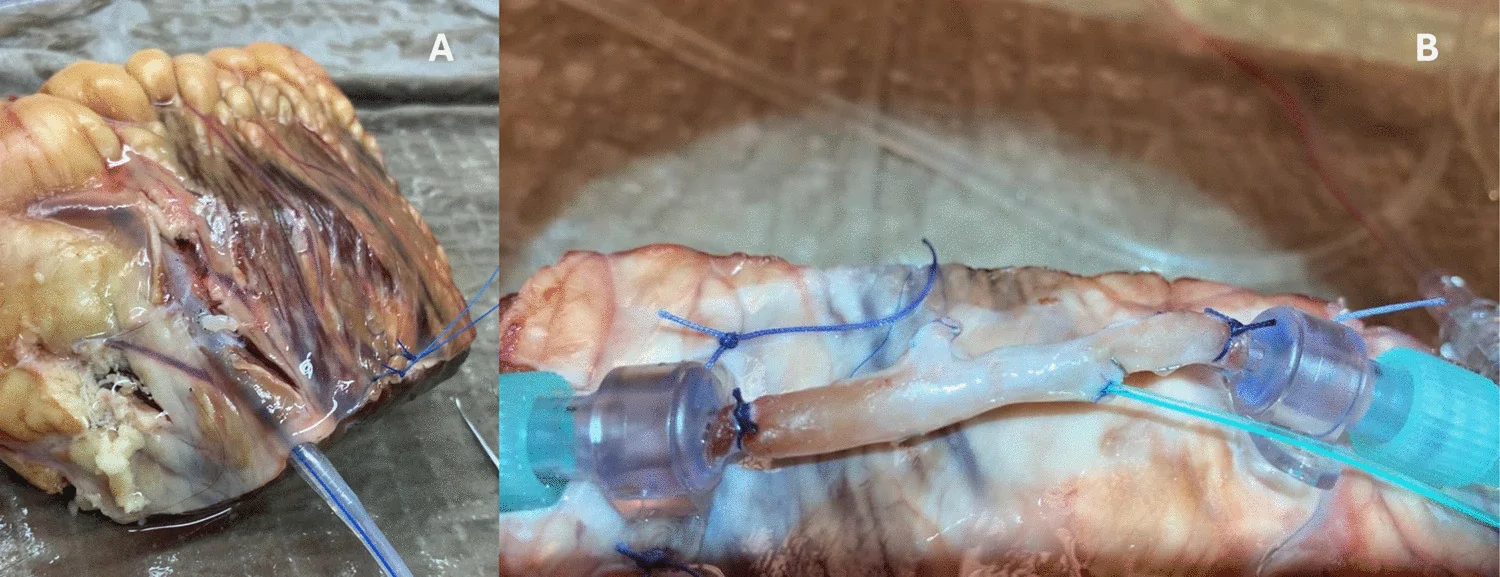
Examples of applications of the pulsatile pump model for realistic microsurgical training, here applying bovine coronary vessels. *Panel A* employs a Foley catheter with inflated balloon inserted into the left anterior descending artery of a bovine specimen – an aneurysm model has been created by suturing in a ligated small vessel. *Panel B* demonstrates the usual setup with two connectors (any connector can be used; here we employed external ventricular drain (EVD) connectors with ligatures) between which any type of synthetic or biological training vessel can be inserted. Both setups can be employed with our pulsatile pump model. Pressure can be monitored either as described within our model and Fig. 2**/**Video 1 or alternatively—as shown in *Panel B* here—by inserting an arterial catheter directly into the training vessel
10. Our instructive Video 1 demonstrates the setup and the necessary steps in detail, so that readers can easily set up the model, which takes around 10 min in total.

## Relevant surgical anatomy

Not applicable.

## Description of the technique

The minimum selection of tools (Table 1) necessary for creating the PPM (Fig. 1): two adjustable infusion pumps (for example Infusomat Space, B. Braun, Melsungen, Germany), one transducer for continuous pressure monitoring (usually for an arterial line or central venous line, for example Combitrans, B. Braun, Melsungen, Germany), one monitor for displaying the pressure and pulsatile rate, one monitoring set, three bags of saline or Ringer’s solution (500 mL or 1000 mL size), two intravenous infusion/drip sets (for example Intrafix set, B. Braun, Melsungen, Germany), one additional three-way stopcock (for example Discofix C, B. Braun, Melsungen, Germany), a syringe of 18G size, a Heidelberger extension line, and two pressure cuffs.

**Table 1:** Components necessary for the pulsatile pump model and approximate costs if new-bought. Please note that all materials are usually available in necessary quantities in hospital settings

Component	Description	Estimated Cost (per unit)
Pulsatile Infusion Pump (Infusomats)	e.g. Infusomat Space (B. Braun, Melsungen, Germany)	€ 272.60
Monitoring Set	Includes transducer (e.g. Combitrans, B. Braun, Melsungen, Germany) and display systems	€ 1,000–€5,000
Intrafix Sets	IV administration sets (2 required)	€ 3.00–€5.00 each
Fluid Bags	3 required. 0.9% NaCl or Ringer solution (500 mL or 1000 mL bottle)	€ 1.00
Three-Way Stopcock	e.g. Discofix C (B. Braun, Melsungen, Germany)	€ 4.99
Heidelberger Extension Line and 18G needle	30 cm length, 18G needle	€ 1.04
Pressure Cuffs	Used for infusion therapy (2 required)	€ 20–€50

The instructional video (Video 1) explains, along with Fig. 2, the setup of the PPM. Basically, first, a conventional pressure monitoring system is used and connected to a three-way stopcock and monitor. Then, a fluid bag with pressure cuff is connected first to a pulsatile infusion pump that is used on many wards, and then to the stopcock, too. Using the hand pump on the fluid bag, the systemic mean arterial pressure (MAP) can be set reliably. Using the setting on the pulsatile infusion system, flow rates and pulsation frequency can be controlled. The stopcock is then connected to the training vessel, which ends in a fluid return line that fills up a backup fluid bag. When the first fluid bag is depleted, it can be simply exchanged with the now filled backup fluid bag. This enables a safe reuse of the (perhaps contaminated in the setting of placental models) fluid in a continuous loop model.

In Fig. 3, we demonstrate the relationship of the pulsation frequency produced by the pulsatile pump depending on the flow rate entered into the infusion pump. A setting of flow rate of 600 mL/h equals approximately a frequency of 60 bpm, 900 mL/h approximately 90 bpm, and 1200 mL/h approximately 120 bpm using our setup. The pulse width is around ± 10 mmHg around the MAP value, in our setup, but depends on the diameters and compliance of the employed materials.

## Indications

It is important to perform microsurgical training in a near-realistic fashion if possible. Traditionally, training under realistic conditions with pulsatile continuous flow is performed using animal models. However, in recent times, understandably the focus of much of the research in microsurgical training has been to reduce or even completely replace the use of (live) animal models. It has meanwhile become standard practice to implement alternative vessels (either synthetic vessels or biological alternatives such as placental vessels or biological vessels such as chicken leg arteries or bovine coronary arteries, for example). Cryopreserved vessels can also be used, reducing the need for live animals further. To date, the use of animals for microsurgical practice is discouraged for ethical and economic reasons; therefore, models and simulators have been progressively catching the interest of trainers and industries.

To achieve pulsatile and continuous flow conditions in these ex-vivo models, a pump is necessary. In the literature, most authors used irrigation or membrane pumps or no pumps at all. In those models, it was not possible to adjust the frequency or the pressure [1–4]. Joseph et al. [5] used a custom-designed pulsatile pump with the ability to adjust frequency and pressure, but it is not described how the pulsatile pump is built. There are a vast variety of highly realistic surgical simulators/perfusion systems, which are not available to everyone, because of high costs, as are most of the other pump systems that come out-of-the-box. This is especially relevant in low-resource settings such as low-to-middle-income countries (LMIC), where microsurgical training is – obviously – just as important.

Therefore, our goal was to create a model, which is easy to build by anyone with access to medical equipment i.e. working in a hospital, without added costs and without modifications to hospital equipment necessary (Table 1). While it includes certain components that may be expensive, these are typically available in large numbers ins hospital settings, making the model highly accessible for medical professionals.

Young neurosurgeons can benefit greatly from a PPM combined with a microsurgical training model. A pulsatile training model, which also enables adjusting frequency and pressure, provides a more accurate representation of human physiology and enables the trainee to see the responsiveness to surgical manipulation. Trainees may develop better tactile feedback of how much pressure to apply without damaging delicate tissues, for example in artificial aneurysm (and aneurysm rupture management) models [1, 2].

It is possible to utilize the pulsatile pump system we developed with various training models to enhance the realism and efficacy of vascular microsurgery training as you can see in our short video demonstration and in Fig. 4. Additionally, it contributes to reducing the number of live animals required for training purposes, in the sense of the common goal of 3R (reduce, replace, refine animal models). The ability to adjust flow rates and pressure in a controlled environment ensures precise replication of physiological conditions.

## Limitations

In very low-resource settings, it might not be viable to employ some of the necessary equipment (monitor, infusion pulsatile pump) from wards. However, the possibility to do so still compares favourably with the alternative, which would be buying off-the-shelf pumps which usually are expensive and, in our experience, also usually fail to produce realistic blood pressure and pulsatility conditions. While introducing a near realistic pulsatile pump model with the ability to adjust the frequency and pressure, it is limited to a maximum frequency of up to around 120 bpm. We did not find a solution for realistic model with a fluid mimicking the coagulation aspects, yet.

## How to avoid complications

If used on a biomaterial model, e.g. cryopreserved vessels, bovine heart, human placenta, it is recommended to ligate all open vessel ends, to avoid loss of pressure and fluid, and to enable a safe closed-loop system. Also, it is important to not use syringes smaller then 18G, because most pumping systems tend not to be able to overcome a higher level of resistance. It is also necessary to start setting the monitoring set up, when the whole circuit is already running. Future iterations of this model could employ two infusion pumps simultaneously—one simulating arterial flow and another venous drainage—to allow realistic simulation of complex vascular scenarios such as arteriovenous malformations.

## Supplementary information

Below is the link to the electronic supplementary material.ESM 1(MP4 519 MB)

## Data Availability

No datasets were generated or analysed during the current study.
